# Bridging the “Valley of Death” in Antifungal Therapy: Next-Generation Biomimetic and Exosome-Inspired Nanocarriers for Invasive Candidiasis

**DOI:** 10.3390/jof12070530

**Published:** 2026-07-19

**Authors:** Bekir Mustafa Yoğurtçu, Ilknur Yilmaz

**Affiliations:** 1 Department of Molecular Biology and Genetics, Graduate School of Science & Engineering, Yildiz Technical University, Istanbul 34220, Turkey; bmustafayogurtcu@gmail.com; 2 Department of Molecular Biology and Genetics, Faculty of Engineering and Natural Sciences, İstanbul Atlas University, Istanbul 34408, Turkey

**Keywords:** biomimetic nanocarriers, invasive candidiasis, fungal biofilms, multidrug resistance, antifungal nanomedicine

## Abstract

Invasive candidiasis, predominantly driven by multidrug-resistant Candida species and intractable biofilms, represents an escalating global health crisis with mortality rates rivaling major infectious diseases. The clinical efficacy of conventional antifungal agents—azoles, polyenes, and echinocandins—is severely compromised by poor tissue penetration, dose-limiting systemic toxicity, and the rapid evolution of complex resistance mechanisms. Here, we review the two-decade structural evolution of nanotechnological interventions designed to overcome these pharmacological and biological barriers. We systematically analyze advanced nanosystems, including lipid-based formulations, natural polymers, and biogenic metallic nanostructures, highlighting their capacity to penetrate the dense extracellular polymeric substance (EPS), combat potential fungal ‘nano-resistance’, and significantly reduce metabolically dormant persister cell populations. The literature search was performed using the electronic databases PubMed, Scopus, Web of Science, and Google Scholar. Publications indexed between 2015 and 2025 were primarily considered, while seminal studies published before 2015 were included when necessary to provide historical context and foundational knowledge. We place specific emphasis on next-generation biomimetic and exosome-inspired nanocarriers, which significantly reduce systemic host toxicity while maximizing targeted antifungal efficacy. In this context, the synergistic integration of smart nanocarriers to actively disassemble fungal resistance networks, such as the target of rapamycin (TOR) signaling pathway and sphingolipid biosynthesis. Finally, we outline a strategic roadmap to bridge the translational “Valley of Death”. By prioritizing manufacturing standardization, comprehensive long-term biosecurity profiling, and rationally designed biomimetic platforms, we propose an alternative way to outpace the evolutionary adaptations of fungal pathogenesis and translate these innovations into the clinic.

## 1. Introduction

The clinical landscape of infectious diseases in the 21st century has been increasingly defined by the silent but devastating surge of invasive fungal infections (IFIs), with Candida species emerging as the most prevalent and challenging opportunistic pathogens [1]. Causing a spectrum of diseases ranging from superficial mucocutaneous infections (such as oral and vulvovaginal candidiasis) to life-threatening disseminated candidemia, these specific pathogens and their resilient biofilm architectures form the primary focus of this review [2]. Invasive candidiasis is estimated to cause approximately 400,000–600,000 cases annually worldwide, with attributable mortality rates ranging from 30% to 60%, making it one of the leading causes of death among IFIs [3], yet fungal diseases remain disproportionately neglected in public health funding. The rising incidence of these infections is intrinsically linked to the growing population of immuno-compromised individuals. Advanced medical interventions, such as intensive chemotherapy for cancer, organ and bone marrow transplantation, and the use of broad-spectrum antibiotics, have created an environment where opportunistic fungi can thrive. Furthermore, the rise of neonatal intensive care has placed preterm infants at high risk for systemic fungal colonization. Historically, *Candida albicans* have been the primary causative agent; however, the last two decades have seen a significant epidemiological shift toward non-albicans Candida (NAC) species, including *Nakaseomyces glabrata* (formerly *Candida glabrata*), *Candida parapsilosis*, *Candida tropicalis*, and the highly concerning *Candidozyma auris* (formerly *Candida auris*). Many of these NAC species exhibit intrinsic resistance or rapidly acquire resistance during treatment, fundamentally challenging our current clinical management [4,5].

The current clinical arsenal for treating candidiasis relies on four primary classes of drugs, each targeting specific components of the fungal cell: (i) Azoles, exemplified by fluconazole, voriconazole, and itraconazole, inhibit the 14-alpha-demethylase enzyme, thereby disrupting ergosterol biosynthesis [6]; while widely employed due to their oral bioavailability, azoles have faced a dramatic decline in efficacy stemming from the widespread emergence of resistance, particularly via efflux pump overexpression [7]. (ii) Polyenes, such as Amphotericin B (*AmB*), act as potent fungicidal agents by binding directly to ergosterol, forming pores that ultimately lead to cell death [6]; despite being the “gold standard” for severe infections, *AmB* is plagued by severe, dose-limiting nephrotoxicity and poor aqueous solubility. (iii) Echinocandins, including caspofungin and micafungin, specifically target the (1,3)-beta-D-glucan synthase enzyme within the fungal cell wall [8]; however, their utility is notably limited by the development of “hotspot” mutations. (iv) Flucytosine, frequently utilized in combination therapies, interferes with DNA and protein synthesis but suffers from a narrow therapeutic window and rapid resistance development [9].

The efficacy of these conventional drugs is further hampered by their inability to penetrate the physiological barriers of the host and the structural defenses of the pathogen, necessitating high doses that increase systemic toxicity. The robust resistance of Candida infections is largely attributed to the pathogen’s ability to transition into complex, multicellular communities known as biofilms. Biofilms are encased in a self-produced EPS or matrix, which acts as a multifunctional shield [10]. This matrix, composed of polysaccharides, extracellular DNA (eDNA), proteins, and lipids, physically sequesters antifungal molecules preventing them from reaching the fungal cells in therapeutic concentrations [11]. However, a significant challenge remains in the global dissemination of these high cost nanotechnological interventions. While clinical need is universal, the socioeconomic barriers in low-to-middle-income countries—where the burden of fungal diseases is often highest—may limit the practical deployment of these advanced platforms. Future strategies must ensure that the transition from bench to bedside includes a framework for global accessibility.

## 2. The Nanotechnology Paradigm: Two Decades of Structural Evolution

Over the last twenty years, nanotechnology has provided a revolutionary platform for overcoming the formidable pharmacological and biological hurdles associated with conventional antifungal therapies. By engineering materials at the nanoscale—typically defined within the 1 to 100 nm size range—researchers have successfully developed “smart,” dynamic delivery systems [12]. These advanced architectures are rationally designed to seamlessly navigate the complex physiological environment of the mammalian host while simultaneously penetrating the robust defensive barriers deployed by fungal pathogens. The inherent physicochemical properties of these nanomaterials, including their high surface-area-to-volume ratio and tunable surface thermodynamics, allow for unprecedented interactions at the cellular and molecular levels. Accordingly, nanotechnology offers several critical, multi-dimensional advantages that fundamentally redefine the modern antifungal therapeutic repertoire:

(i) Enhanced Solubilization and Bioavailability: A significant proportion of classical antifungal agents, most notably the polyene *AmB* and advanced triazoles like voriconazole, suffer from severe intrinsic hydrophobicity [13]. This poor aqueous solubility leads to erratic absorption profiles, rapid degradation in systemic circulation, and suboptimal bioavailability at the site of infection. Nanosystems—ranging from liposomal formulations to nanostructured lipid carriers (NLCs) and polymeric micelles—effectively address this bottleneck by encapsulating these hydrophobic molecules within protective, lipophilic cores or amphiphilic matrices [14]. This encapsulation not only shields the active pharmaceutical ingredient (API) from enzymatic and hydrolytic degradation but also dramatically improves its thermodynamic dispersion in the aqueous physiological milieu, ensuring that peak therapeutic concentrations consistently reach the infected tissues [15].
(ii) Targeted Delivery and Biofilm Retention: The structural resilience of Candida biofilms, fortified by an impenetrable EPS, severely restricts conventional drug diffusion [16]. Nanotechnology circumvents this diffusion limitation through sophisticated surface functionalization. By decorating the exterior of nanocarriers with specific biological ligands—such as monoclonal antibodies directed against fungal surface adhesins like the Agglutinin-like sequence 3 (Als3) protein—researchers can achieve highly specific, active targeting of fungal cells. The integration of mucoadhesive and highly polycationic polymers, such as chitosan, is hypothesized to allow these nanosystems to exploit the negatively charged components of the fungal cell wall and the anionic EPS matrix [17,18]. Although in vitro models have demonstrated that electrostatic interactions promote the localized accumulation and prolonged retention of antifungal payloads within the biofilm microenvironment, the effectiveness of this mechanism under complex in vivo conditions—where factors such as high hemodynamic shear stress and rapid protein corona formation are prevalent—has not yet been comprehensively validated [19]. Furthermore, nanoparticle penetration efficacy is not uniform across all stages of biofilm development but is strongly influenced by biofilm maturity, matrix composition, and species-specific architecture. As Candida biofilms mature, the EPS matrix becomes progressively denser and increasingly enriched in structural polysaccharides, particularly β-1,3-glucan. This dense network can act as a physical barrier, limiting nanocarrier diffusion and reducing penetration efficiency compared with early-stage biofilms. In addition, EPS composition varies considerably among Candida species. While the biofilm matrix of *C. albicans* contains substantial amounts of eDNA, polysaccharides, and glycoproteins, non-albicans species such as *N. glabrata* and *C. parapsilosis* often exhibit distinct protein-to-carbohydrate ratios, lipid compositions, and matrix organization [20,21]. These interspecies differences can significantly influence nanoparticle–biofilm interactions and therapeutic performance.
(iii) Species-Specific Biofilm Targeting and EPS Matrix Penetration: The biochemical diversity of the EPS matrix in non-albicans species necessitates species-specific structural considerations in nanocarrier design. For instance, the highly protein- and β-glucan-enriched biofilm matrix of *N. glabrata* severely exacerbates azole resistance by physically sequestering the drug. To breach this fortified barrier, protein-based nanocarriers, such as minocycline-loaded bovine serum albumin nanoparticles (Min-NPs), are strategically employed to effectively reverse resistance and restore azole susceptibility [22]. Similarly, the intractable, eDNA-rich biofilms of *C. auris* can be systematically dismantled using specific inorganic platforms. Biogenic silver nanoparticles (AgNPs) [23] and biomimetically functionalized hybrid systems (e.g., tyrosol-conjugated gold nanoparticles, Chi-TY-AuNPs) successfully penetrate the *C. auris* EPS to eradicate both active hyphae and dormant persister cells through localized oxidative stress (Table 1) [24]. The comprehensive biomimetic mechanisms, intracellular dynamics, and host biocompatibility profiles of these advanced nanosystems are discussed in detail in Section 5 and Section 6.
(iv) Reduced Host Systemic Toxicity: The dose-limiting toxicity of conventional therapies—such as the devastating nephrotoxicity and hemotoxicity intrinsically associated with *AmB*—is a direct consequence of off-target interactions with mammalian cell membranes. Nanocarriers inherently mitigate this through temporally and spatially controlled drug release kinetics [31]. By securely housing the cytotoxic cargo until the carrier reaches the specific infection locus, these “stealth” systems prevent premature systemic exposure. This targeted approach significantly minimizes collateral damage to human renal and hepatic tissues, a protective effect clinically quantifiable by the dramatic reduction in Lactate Dehydrogenase (LDH) leakage. The preservation of baseline LDH levels serves as a definitive biological hallmark of maintained mammalian cellular integrity, thereby safely widening the therapeutic index of traditionally toxic drugs [32].
(v) Synergistic Co-delivery and Resistance Reversal: The rapid emergence of multidrug-resistant (MDR) Candida strains, driven by the massive overexpression of efflux pumps and target-site enzymatic mutations, necessitates combinatorial therapeutic strategies. Novel multi-compartmental nanosystems are uniquely equipped to simultaneously deliver disparate classes of pharmacological agents [33]. For instance, the co-encapsulation of repurposed antibiotics such as minocycline alongside fluconazole within a single nanocarrier allows for synchronized, spatiotemporal delivery [34]. This synergistic action can significantly reverse azole resistance by overwhelming complex fungal defense networks. Additionally, in vitro studies indicate that integrating natural bioactive compounds, such as essential oils and monoterpenes (e.g., carvacrol or linalool), with traditional antifungals can effectively disrupt fungal membrane integrity [35]. It is proposed that this disruption may circumvent active efflux mechanisms, thereby potentially restoring the fungicidal activity of the primary payload against highly resilient phenotypic variants and dormant persister cells [21]. Nevertheless, these mechanistic models require further substantiation through advanced in vivo infection models to confirm their clinical viability (Table 2).

The clinical efficacy of antifungal nanocarriers is closely associated with their intended route of administration, which fundamentally influences the structural and physicochemical characteristics required for optimal biodistribution and therapeutic performance [36]. Intravenous delivery requires highly stable, exosome-inspired or biomimetic nanocarriers capable of evading immune recognition and prolonging systemic circulation to effectively target deep-seated fungal infections. In contrast, localized delivery strategies, such as pulmonary aerosolization for respiratory infections and microneedle-based platforms for dermal or mucocutaneous candidiasis, necessitate carriers engineered for efficient tissue penetration and sustained site-specific drug release. These approaches enhance local therapeutic efficacy while minimizing systemic exposure and associated toxicity [37].

Despite these advantages, the possible emergence of “nano-resistance” must be carefully considered in long-term clinical applications. Similar to classical antifungal resistance, fungal pathogens may adapt through both structural and biochemical mechanisms that reduce nanoparticle efficacy. These adaptations may include cell wall remodeling—such as increased chitin content and enhanced β-1,3-glucan cross-linking—which decreases porosity and limits nanoparticle penetration into biofilms.

In addition, many nanoparticle-based antifungal strategies rely on the induction of reactive oxygen species (ROS) to exert fungicidal effects [30,38,39]. Under sustained sub-lethal exposure, fungi may counteract this stress by upregulating antioxidant defense systems, including enzymes such as superoxide dismutase, catalase, and glutathione peroxidases, thereby neutralizing oxidative damage. They may also enhance intracellular sequestration mechanisms, such as vacuolar trapping of nanoparticles, further reducing their interaction with essential cellular targets [30]. Collectively, these potential adaptations highlight the need for multi-targeted and dynamically designed nanotherapeutic strategies rather than reliance on a single platform. By utilizing hybrid carriers that simultaneously exert mechanical membrane disruption, silence adaptive stress-response genes (e.g., via CRISPR-Cas9 payloads), and deliver traditional pharmacological agents, researchers can ensure that the fungal pathogen is metabolically overwhelmed across multiple cellular fronts before compensatory evolutionary adaptations can take root.

**Table 2:** Comprehensive mechanistic summary of nanosystems for candidiasis (2000–2026).

Nanosystem Type	Key Payloads/Surface Modifications	Primary Mechanistic Advantage	Specific Clinical/Experimental Findings	References
**Solid Lipid Nanoparticles (SLN)**	Fluconazole	Enhanced stability and intracellular accumulation.	Restores fluconazole efficacy in MDR *Candida* isolates; lower MICs than free drug.	[26]
**Nanostructured Lipid Carriers (NLC)**	Fluconazole + Monoterpenes (Carvacrol, Linalool)	High drug loading; synergistic EPS disruption.	4–8-fold increase in fluconazole activity; spherical shape (144.5 nm).	[40]
**Nanoemulsions (NE)**	Amphotericin B (*AmB*) + Eucalyptol	High tissue/skin penetration; sustained cargo release.	10× deeper penetration in topical models; reduced hemotoxicity and LDH release.	[41]
**Tyrosol-Functionalized Gold NPs (Chi-TY-AuNPs)**	Tyrosol + Chitosan Coating	Antifouling; ROS generation; gene silencing.	Substantially disrupts mature biofilms; downregulates some biosynthesis genes, excluding ERG11.	[25]
**Biogenic Silver NPs (AgNPs)**	Biogenic capping agents/No additional payload	Low environmental toxicity; multi-target action.	Potent activity against *Candida* spp. with minimal damage to human host cells.	[42]
**Albumin Nanoparticles**	Minocycline + Fluconazole	Biocompatibility; synergistic reversal of resistance.	Successful eradication of azole-resistant *N. glabrata* at clinically safe doses.	[22]
**Farnesol-Loaded Liposomes**	Quorum-sensing molecule Farnesol	Inhibition of filamentation and biofilm formation.	Synergistically enhances fluconazole efficacy against resistant biofilms.	[43]

## 3. Expanding the Payload: Nanocarriers for Gene-Modulating Antifungal Therapies

Over the past two decades, research has generated a diverse range of nanosystems tailored to specific clinical needs in antifungal therapy. Lipid-based nanosystems—including liposomes (e.g., *AmB*isome), solid lipid nanoparticles (SLNs), and NLCs—have demonstrated the ability to enhance drug solubility, stability, and intracellular delivery [44]. In parallel, polymeric nanoparticles based on natural polymers like chitosan offer intrinsic antimicrobial [45] and mucoadhesive properties, while metallic and inorganic nanoparticles, including silver (Ag) and gold (Au), exhibit intrinsic antifungal activity, primarily through the generation of ROS. However, the industrial viability and scalability of these systems vary significantly, necessitating rigorous comparative studies on their cost-effectiveness and batch-to-batch reproducibility under Good Manufacturing Practice (GMP) standards [46].

**Figure 1:**
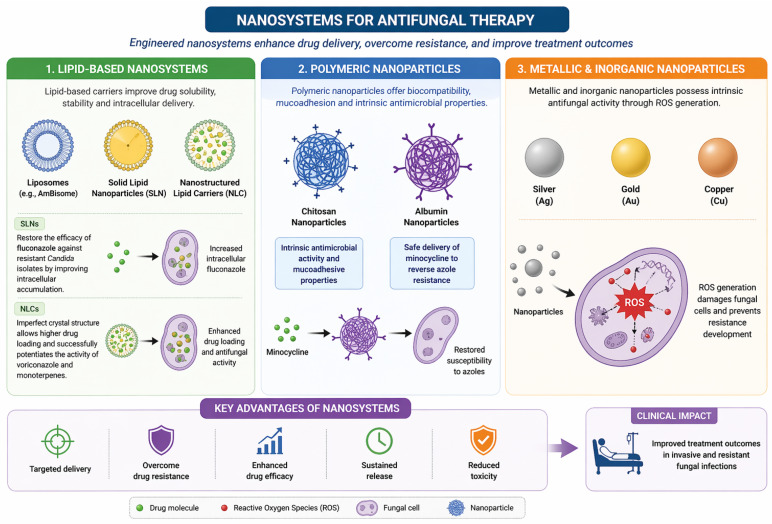
Nanosystems for antifungal therapy: classification, mechanisms, and therapeutic impact. Lipid-based nanosystems (liposomes, SLNs, NLCs) enhance drug solubility, stability, and intracellular delivery, restoring antifungal efficacy against resistant *Candida* isolates. Polymeric nanoparticles (e.g., chitosan and albumin) provide intrinsic antimicrobial and mucoadhesive properties and enable safe drug delivery to overcome azole resistance, while metallic nanoparticles (Ag, Au, Cu) exert antifungal effects primarily through ROS generation. Together, these nanosystems improve drug loading, targeting, and therapeutic outcomes while reducing toxicity in resistant fungal infections.

While the generation of ROS by metallic and inorganic nanoparticles provides a potent, multi-targeted mechanism for fungal cell destruction, this approach inherently risks off-target oxidative damage to mammalian tissues [47,48]. Uncontrolled ROS production can trigger lipid peroxidation, protein denaturation, and DNA damage in host cells, significantly narrowing the therapeutic window. To maintain a safe balance between antifungal efficacy and host biocompatibility, these nanocarriers should be specifically engineered [49,50]. By utilizing charge-balancing ligands or biomimetic, exosome-inspired cloaking, researchers can ensure that the oxidative burst is selectively catalyzed only upon interaction with the specific microenvironment of the fungal biofilm, thereby preserving the integrity of surrounding mammalian cells. Additionally, metallic and inorganic nanoparticles, including silver (Ag), and gold (Au) exhibit intrinsic antifungal activity, primarily through the generation of ROS, leading to effective fungal cell destruction [51] (Figure 1).

As these nanocarrier platforms mature, their structural utility is expanding beyond the passive delivery of conventional small-molecule antifungals to the transportation of highly complex, sensitive biological macromolecules [52,53]. A prime example of this evolution is the ongoing effort to deliver gene-modulating payloads, such as CRISPR-Cas9 ribonucleoproteins (RNPs). Unlike small hydrophobic drugs, CRISPR-Cas9 constructs are large, highly susceptible to enzymatic degradation, and face profound physical barriers when attempting to penetrate the dense EPS of fungal biofilms [54]. To overcome these delivery barriers, advanced biomimetic architectures, particularly exosome-like nanovesicles, are currently being investigated as protective carriers for these delicate genetic payloads (Table 3). These specialized nanocarriers are hypothesized to shield the CRISPR-Cas9 cargo from premature degradation, holding the potential to facilitate targeted delivery directly into dense fungal biofilms. Rather than viewing genome editing solely as a genetic mapping tool, its integration into nanomedicine underscores the exceptional payload capacity and versatility of biomimetic carriers [55]. If successfully validated in future clinical studies, this convergence could theoretically shift nanotechnology from a purely passive delivery platform into an active, gene-modulating strategy capable of silencing key resistance pathways—such as the TOR signaling cascade or sphingolipid biosynthesis—prior to conventional antifungal treatment [56].

However, while these nanocarrier-mediated delivery strategies offer highly promising mechanistic insights in vitro, their direct translational applicability remains largely theoretical at this stage. Genetically engineered knockouts and payload delivery evaluated in planktonic cultures or static in vitro biofilms frequently fail to capture the dynamic complexity of in vivo fungal infections [57,58]. In a true physiological context, the dense EPS matrix, high hemodynamic shear stress, and active host immune responses create a highly heterogeneous microenvironment that may severely alter the penetration kinetics of these large, macromolecule-loaded nanoparticles. Therefore, transitioning from conceptual in vitro payload delivery to actual clinical efficacy will require advanced in vivo infection models capable of accurately simulating the robust three-dimensional architecture and evolutionary resilience of clinical biofilms [59,60,61].

**Table 3:** Comparative analysis of conventional antifungal therapies and nano-enabled delivery systems (2000–2026).

Parameter	Conventional Antifungal Agents (Azoles, Polyenes, Echinocandins)	Nano-Enabled Antifungal Systems	Ref.
**MIC**	Often require higher drug concentrations due to poor penetration into biofilms and efflux-mediated resistance.	Frequently demonstrate 2–10-fold MIC reduction through enhanced intracellular delivery and improved biofilm penetration.	[24]
**Biofilm Penetration**	Limited penetration through the EPS matrix.	Nanoparticles (<100–200 nm) efficiently penetrate EPS barriers, enabling localized drug accumulation and sustained release within biofilm structures.	[12,62]
**Systemic Toxicity**	High-dose therapy often causes nephrotoxicity, hepatotoxicity, and hemotoxicity, particularly with amphotericin B.	Encapsulation reduces off-target exposure and minimizes LDH leakage, renal injury, and hepatic toxicity.	[63,64]
**Pharmacokinetic** **Properties**	Poor aqueous solubility, rapid clearance, limited tissue distribution, and short circulation half-life.	Improved solubility, prolonged circulation, controlled drug release, enhanced tissue accumulation, and greater bioavailability.	[65,66]
**Resistance Overcoming** **Capability**	Frequently compromised by efflux pump overexpression, target-site mutations, and biofilm-associated tolerance mechanisms.	Co-delivery strategies, ROS-mediated fungal killing, efflux pump circumvention, and multi-target mechanisms effectively restore antifungal activity against resistant strains.	[67,68]
**Drug Stability**	Susceptible to degradation and loss of activity during systemic circulation.	Nanocarriers protect payloads from enzymatic degradation and premature release, enhancing therapeutic stability.	[69,70]
**Targeted Delivery**	Primarily passive distribution with limited infection-site specificity.	Surface-functionalized nanoparticles and biomimetic carriers enable active targeting and retention at infection sites.	[71,72]
**Persister Cell** **Eradication**	Limited efficacy against metabolically dormant persister cells embedded within biofilms.	Metallic nanoparticles, hybrid systems, and sustained-release formulations effectively target persister populations through ROS generation and prolonged exposure.	[73,74]
**Therapeutic Index**	Narrow therapeutic window due to dose-limiting toxicity.	Expanded therapeutic index resulting from improved efficacy at lower doses and reduced systemic toxicity.	[75]
**Clinical Translation** **Status**	Multiple FDA/EMA-approved agents currently used in clinical practice.	Several formulations (e.g., liposomal amphotericin B) are clinically approved, while advanced biomimetic and exosome-inspired systems remain in preclinical or early translational development.	[76]

By systematically knocking out specific efflux pump genes (e.g., *cdr1Δ/cdr1Δ* in *C. albicans*), researchers can determine whether nanocarriers truly evade efflux systems or merely overcome them through increased intracellular concentrations [77]. Moreover, exosome-like nanovesicles are emerging not only as drug delivery vehicles but also as protective carriers for CRISPR–Cas9 RNPs, holding the potential to enable targeted delivery into dense fungal biofilms, though extensive in vivo validation remains necessary [78]. This convergence shifts nanotechnology from a passive delivery platform to an active, gene-modulating strategy capable of silencing key resistance pathways—such as the TOR signaling cascade or sphingolipid biosynthesis—prior to conventional antifungal treatment [79].

However, while these CRISPR-Cas9-mediated in vitro investigations provide invaluable mechanistic insights, they present significant limitations that complicate their direct translational applicability. Genetically engineered knockouts evaluated in planktonic cultures or static in vitro biofilms frequently fail to capture the dynamic complexity of in vivo fungal infections [80]. In a true physiological context, the dense EPS matrix, high hemodynamic shear stress, and active host immune responses create a highly heterogeneous microenvironment that can alter both nanoparticle penetration kinetics and fungal gene expression profiles [81]. Furthermore, the targeted gene silencing observed in highly controlled laboratory settings may be circumvented by alternative, compensatory metabolic pathways when the pathogen is subjected to in vivo host pressures. Therefore, transitioning from in vitro genomic mapping to actual clinical efficacy requires advanced in vivo infection models that can accurately simulate the robust three-dimensional architecture and evolutionary resilience of clinical biofilms.

## 4. Bridging the Gap: Overcoming the “Valley of Death”

Despite the significant in vitro success of advanced nanosystems in mitigating and reducing resistant fungal pathogens, the arduous transition from the laboratory bench to the clinical bedside remains a profound translational bottleneck [82]. This formidable developmental chasm—widely recognized in pharmacological and biomedical engineering as the “Valley of Death”—is characterized by a staggering clinical attrition rate, where highly promising, rationally designed nanotherapeutics consistently fail to demonstrate real-world viability [83]. The discrepancy between benchtop efficacy and human physiological reality stems from a myriad of complex biological, manufacturing, and regulatory challenges that must be systematically deconstructed to actualize the potential of antifungal nanomedicine (Table 4).

First, the fundamental disconnect between static in vitro assays and the dynamic in vivo environment cannot be overstated. Traditional laboratory biofilm models categorically fail to replicate the extreme hemodynamic shear stresses, the rapid formation of the biomolecular protein corona (opsonization), and the aggressive clearance mechanisms of the host’s mononuclear phagocyte system (MPS) [84]. A nanocarrier that demonstrates significantly reduced Candida biofilms in a highly controlled microtiter plate is frequently neutralized by immune surveillance or prematurely degraded by serum enzymes before it can penetrate the deep-seated infectious nidus [85]. Traversing this biological gap requires a paradigm shift toward biomimetic architecture such as, erythrocyte-cloaked or exosome-inspired carriers, that are engineered to improve biological precision and theoretically minimize detection by hostile systemic barriers, although achieving complete immune evasion in human clinical models remains a significant future challenge [86].

**Table 4:** Nanomedicine strategies for antifungal therapy (2016–2026): a comparative assessment of design, efficacy, and clinical translation.

Nanosystem Type	Primary Antifungal Cargo and Typical Dose/Combination	Nm	PDI	mV	EE%	Antifungal Efficacy	Translational Features	Ref.
Albumin Nanoparticles (Min-NPs)	Minocycline + Fluconazole& (In vivo: 5 mg/kg FLZ + 2.5 mg/kg MIN)	~180–220	0.19 ± 0.02	−15 to −25	FLZ: ~82% MIN: ~75%	Reversed azole resistance; sterilized renal fungal burden in vivo	Excellent biocompatibility; minimal hepatotoxicity/nephrotoxicity; protein instability during storage remains a challenge; advanced preclinical stage	[22]
Polymer–Metal Hybrid Nanoparticles (Chi-TY-AuNPs)	Tyrosol + Gold Core and (In vitro: 50–100 µg/mL)	10.34	0.29 ± 0.01	+45.5	46.08%	Mature biofilm degradation; suppression of *FKS1* and *ERG* genes	Potent antibiofilm activity; uncertain long-term biodistribution of Au core; preclinical stage	[25]
Nanostructured Lipid Carriers (NLCs)	Fluconazole, Voriconazole and (Topical/In vitro: 1–5 mg/mL)	144.5	0.23 ± 0.01	−23.5	85–92%	4–8-fold enhancement of azole efficacy against MDR Candida	Improved drug loading and reduced systemic toxicity; lipid polymorphism may affect shelf life; preclinical to early clinical stage	[87]
Nanoemulsions (NEs)	Essential Oils, AmB and (Topical: 0.1–0.5% *w*/*w*)	80–250	0.20–0.40	−10 to −35	>90%	Up to 10-fold greater tissue penetration in topical models	Simple manufacturing and favorable safety profile; thermodynamic instability during storage; preclinical stage	[88]
Solid Lipid Nanoparticles (SLNs)	Amphotericin B, Fluconazole & (In vivo: 1–5 mg/kg)	120–300	0.45	−15 to −40	50–70%	Reduced MICs and restored azole susceptibility	Improved drug protection; risk of drug expulsion during crystallization; preclinical/early clinical stage	[89]
Exosome-Inspired Nanocarriers	siRNA, CRISPR RNPs, Drugs & (In vitro: 20–50 nM nucleic acids)	50–200	0.15–0.25	−5 to −30	40–60%	Enhanced penetration through dense EPS and intracellular delivery	Excellent biocompatibility and immune evasion; GMP-scale production remains challenging; proof-of-concept to early preclinical stage	[90]
Liposomes	Amphotericin B& (Clinical: 3–5 mg/kg/day)	80–250	≤0.15	−10 to −30	80–99%	Enhanced efficacy with reduced nephrotoxicity	Most clinically mature nanocarrier platform; expensive manufacturing; clinically translated	[91]
Polymeric Micelles	Itraconazole, Amphotericin B & (In vivo: 5–10 mg/kg)	20–150	0.10–0.25	−5 to −20	75–85%	Improved solubility and intracellular drug accumulation	Excellent for poorly soluble drugs; dilution instability possible; preclinical stage	[92]
Mesoporous Silica Nanoparticles (MSNs)	Fluconazole, Peptides& (In vitro: 100–250 µg/mL)	50–300	0.20–0.30	−15 to −35	>90%	Sustained release and biofilm penetration	Highly tunable and functionalizable; biodegradation concerns remain; preclinical stage	[93]
Dendrimers	Amphotericin B, Caspofungin & (In vitro: 1–10 µg/mL)	5–20	≈0.10–0.30	+10 to +40	80–95%	Membrane disruption and synergistic fungicidal activity	Multivalent targeting capability; cationic toxicity remains a concern; preclinical stage	[94]
Chitosan Nanoparticles	Fluconazole, Essential Oils & (In vivo: 5–20 mg/kg)	50–400	≈0.20–0.40	+20 to +60	55–75%	Intrinsic antifungal activity and potent biofilm inhibition	Biodegradable, mucoadhesive and low-cost; batch variability may affect reproducibility; advanced preclinical stage	[95]
Silver Nanoparticles (AgNPs)	Silver Ions (Intrinsic Cargo) & 5–100 μg/mL	5–100	≈0.15–0.35	−20 to +30	N/A	Broad-spectrum fungicidal activity and biofilm eradication	Effective against MDR strains; concerns regarding cytotoxicity and environmental accumulation; preclinical stage	[96]
Metal–Organic Frameworks (MOFs)	Amphotericin B, Fluconazole, Photosensitizers& Dosage: N/A	50–250	≈0.15–0.30	−10 to +20	>95%	Stimuli-responsive release and ROS-mediated fungal killing	Extremely high loading capacity; regulatory pathway remains unclear; early preclinical stage	[97]
Nanogels	Amphotericin B, Miconazole, Clotrimazole& Dosage: N/A	50–300	0.26 ± 0.03	−15 to +20	89.5%	Sustained local release and prolonged mucosal retention	Particularly attractive for oral and vaginal candidiasis; limited clinical data; preclinical stage	[98]
Cell-Membrane-Coated Nanoparticles	Amphotericin B, Fluconazole, Antimicrobial Peptides& Dosage: N/A	80–250	0.25–0.35	N/A	75–95%	Enhanced fungal targeting and immune evasion	Biomimetic delivery with prolonged circulation; manufacturing complexity remains a bottleneck; proof-of-concept stage	[99]

Abbreviations: NLCs, nanostructured lipid carriers; SLNs, solid lipid nanoparticles; MSNs, mesoporous silica nanoparticles; MOFs, metal–organic frameworks; EPS, extracellular polymeric substance; MDR, multidrug-resistant; ROS, reactive oxygen species; EE, encapsulation efficiency. nm: Particle Size, mV: Zeta Potential, PDI: Polydispersity Index, N/A: Not Applicable.

Second, the industrial manufacturing scale-up of these intricate, multi-component nanosystems poses a massive and frequently underestimated engineering hurdle. Synthesizing precisely functionalized nanoparticles at the milligram scale under strictly controlled academic conditions is vastly different from achieving industrial production compliant with stringent GMP standards [100]. The commercial and clinical translation of highly complex hybrid structures is frequently derailed by insurmountable issues regarding batch-to-batch reproducibility, thermodynamic instability over extended shelf lives, and the precise maintenance of a uniform polydispersity index. Nanoplatforms burdened by overly convoluted, multi-step synthesis protocols are destined to fail during translational scale-up. Therefore, clinical prioritization must pivot toward streamlined, high-yield manufacturing processes that utilize biodegradable, scalable materials without sacrificing targeted efficacy [101]. Furthermore, the regulatory pathway for multi-component nanotherapeutics remains complex. Current FDA and EMA frameworks require exhaustive characterization of each individual component within a hybrid system. Bridging the ‘Valley of Death’ therefore requires not just engineering innovation, but a proactive alignment with regulatory safety standards early in the design phase (Table 5).

This translational barrier is particularly pronounced for metallic and inorganic nanoparticles. Over long-term storage, these inorganic cores are highly susceptible to physical and chemical destabilization, such as irreversible agglomeration, Ostwald ripening, and oxidative degradation [107]. These phenomena drastically skew their uniform polydispersity index (PDI) and compromise their specific surface plasmon resonance or ROS-generating properties. Furthermore, the thermodynamic instability of their complex surface functionalization—where essential biomimetic capping agents or charge-balancing ligands may detach or degrade over extended shelf lives—rapidly leads to a loss of colloidal stability. Aligning the production of these metallic systems with strict GMP standards introduces further complications [108]. Traditional batch-reactor synthesis methods fail to maintain the exact temporal control over nucleation and growth phases required to produce uniformly sized metallic cores on a commercial scale. To overcome these persistent manufacturing hurdles, industrial progression must pivot toward automated, continuous-flow technologies, such as microfluidic synthesis platforms, to drastically reduce intra-batch variance. Additionally, establishing robust preservation strategies, including optimized lyophilization protocols utilizing specific cryoprotectants, is indispensable for preventing aggregation and ensuring the long-term structural integrity of these advanced inorganic nanotherapeutics [109].

A notable exception exists in the realm of topical drug delivery, where formulations face fewer systemic regulatory hurdles. For example, a randomized controlled clinical trial evaluating fluconazole-loaded SLNs formulated into a topical gel demonstrated superior therapeutic efficacy and faster symptom resolution in patients compared to conventional commercial creams. However, for invasive candidiasis, clinical data is markedly limited. Systematic reviews evaluating advanced platforms, such as silver nanoparticles directed against *Candida albicans*, reveal that the overwhelming majority of studies remain strictly in vitro, with a stark absence of robust systemic human trials [110,111]. The current clinical landscape for invasive fungal infections is therefore still dominated by first-generation lipidic systems, such as liposomal amphotericin B. This disparity highlights the urgent need for accelerated translational research focused on standardizing the large-scale manufacturing and chronic safety profiling of these advanced biomimetic and exosome-inspired carriers, enabling them to progress from promising laboratory models to active clinical investigation [112].

Additionally, engineering these platforms to possess long-term thermodynamic stability at room temperature—such as through optimized lyophilization protocols—is essential. Eliminating the strict requirement for expensive, uninterrupted cold-chain logistics will drastically reduce distribution costs and facilitate practical deployment in resource-limited clinical settings. Ultimately, bridging the translational ‘Valley of Death’ requires not only scientific ingenuity but also proactive alignment with global health initiatives, fostering open-source technology transfer, and establishing strategic manufacturing partnerships to ensure equitable, worldwide access to antifungal nanomedicines [12]. The regulatory landscape governing next-generation nanomedicines requires comprehensive, longitudinal safety profiles, including evaluation of chronic immunogenicity and long-term toxicity, extending beyond baseline acute cytocompatibility metrics. Establishing genuine therapeutic safety requires rigorous in vivo evaluation of chronic immunogenicity, biodistribution, and long-term toxicity [113].

For nanocarriers to achieve clinical translation and regulatory approval, in vitro efficacy alone is insufficient. Regulatory agencies require rigorous quantitative validation of several critical physicochemical parameters. Among these, particle size distribution and the PDI should ideally remain ≤0.2 to ensure uniformity, predictable biodistribution, and reduced off-target toxicity [114]. During the industrial scale-up of nanomedicines, maintaining a narrow PDI is essential to overcome manufacturing challenges like spatial-temporal gradients and sterile filtration clogging [115]. Therefore, a tight PDI is not merely a regulatory checkpoint but a fundamental technical requirement to guarantee long-term thermodynamic stability, predictable pharmacokinetics, and consistent batch-to-batch therapeutic efficacy [116]. In addition, colloidal stability and an appropriately optimized zeta potential are essential to prevent nanoparticle aggregation while enabling safe circulation within biological systems. Equally important are well-characterized and mathematically predictable drug-release kinetics, which minimize the risk of burst release–associated toxicity and ensure the controlled, sustained delivery of therapeutic agents to the target biofilm matrix. Collectively, these parameters are fundamental determinants of nanocarrier safety, efficacy, and regulatory acceptance [117,118].

## 5. Exosome-Inspired Delivery and Biomimetic Carriers

The pharmacological limitations of conventional antifungal therapeutics, compounded by the escalating crisis of multidrug resistance (MDR), have necessitated a paradigm shift toward advanced nanocarrier systems capable of penetrating the complex defensive barriers of fungal pathogens [119]. Among exosome-inspired delivery systems, mesenchymal stem cell (MSC)-derived EVs have emerged as the most clinically relevant and biologically stable source of natural nanocarriers. In contrast to vesicles derived from immortalized cell lines, MSC-derived exosomes exhibit superior immunological compatibility, intrinsic tropism toward inflamed and infected tissues, and a highly conserved lipid–protein composition that enhances their systemic stability and circulation half-life. These properties make MSC-derived exosomes particularly attractive templates for engineering biomimetic antifungal delivery systems with improved in vivo translational potential [120]. To properly contextualize the therapeutic utility of immunologically compatible MSC-derived carriers, it is crucial to contrast them with the natural EVs produced by fungal pathogens. While biomimetic engineering aims to create ‘stealth’ delivery systems, naturally occurring fungal EVs are profoundly immunogenic. For example, EVs derived from pathogenic dimorphic fungi, such as *Talaromyces marneffei*, are actively utilized by the pathogen to manipulate host immune responses [121]. *T. marneffei* yeasts secrete lipid-bilayer EVs loaded with bioactive protein components that are readily internalized by host macrophages, triggering a potent pro-inflammatory cascade. Unlike the immune-evasive design of MSC-exosomes, these fungal vesicles significantly elevate the secretion of inflammatory cytokines, highlighting their intrinsic role in pathogenesis and host-pathogen communication [122]. This stark functional dichotomy underscores why therapeutic nanocarriers must be meticulously engineered—using host-derived mammalian templates like MSCs—to avoid the robust immune recognition and rapid systemic clearance naturally provoked by fungal extracellular components [104,123].

**Figure 2:**
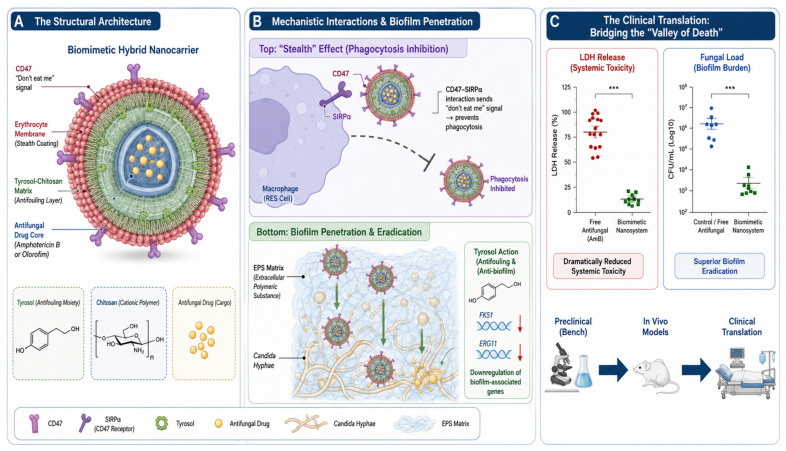
Biomimetic hybrid nanocarrier for antifungal therapy and biofilm disruption. (**A**) Schematic of a biomimetic nanocarrier composed of an antifungal drug core encapsulated within a tyrosol–chitosan matrix and cloaked with an erythrocyte membrane expressing CD47 for immune evasion. (**B**) The nanocarrier exploits CD47–SIRPα signaling to avoid phagocytosis and penetrate fungal biofilms, where tyrosol mediates antifouling activity and downregulates biofilm-associated genes. (**C**) In vivo translation demonstrates reduced systemic toxicity and enhanced biofilm clearance compared with free antifungal treatment. Collectively, this platform bridges preclinical and clinical application by improving therapeutic efficacy while minimizing host toxicity. (*** denotes *p* < 0.001).

The so-called “stealth” behavior of exosomes is therefore not merely a structural feature but a biologically programmed function originating from their parental MSC microenvironment, which equips these vesicles with surface proteins (including CD47-like signaling modulators and integrin profiles) that actively reduce macrophage recognition and facilitate immune evasion in systemic circulation [124] (Figure 2).

Beyond their role in small-molecule and protein delivery, MSC-derived exosomes are increasingly being investigated as non-viral vectors for the transport of nucleic acid cargo, including plasmid DNA and CRISPR-associated genetic constructs. Their natural lipid bilayer architecture enables efficient encapsulation of plasmid DNA while protecting it from nuclease degradation, thereby providing a biocompatible platform for gene modulation strategies aimed at silencing fungal resistance pathways such as efflux pump overexpression and biofilm-associated gene networks. This dual functionality is hypothesized to transform MSC-derived exosomes from passive drug carriers into active genetic intervention systems, positioning them at the intersection of nanomedicine and fungal functional genomics. Among protein-based biomimetic platforms, albumin-derived nanoparticles represent a broadly applicable class of clinically translatable carriers, with bovine serum albumin (BSA)-based systems serving as a well-characterized and widely adopted model for encapsulating hydrophilic and amphiphilic antimicrobial agents. Within this framework, minocycline-loaded albumin nanoparticles (Min-NPs) can be regarded as a representative case study illustrating the general advantages of protein scaffolds, including tunable drug-loading capacity, structural stability, and sustained-release behavior [22]. Rather than being interpreted as an isolated formulation, such systems collectively exemplify how protein-based nanocarriers can be engineered to modulate pharmacokinetics and improve intracellular drug bioavailability in resistant fungal infections. The exceptional biocompatibility, robust drug-loading capacity, and structural stability of albumin molecules render them highly advantageous scaffolds for nanocarrier design. During the synthesis of Min-NPs, BSA molecules are pre-treated with dithiothreitol (DTT) to cleave intramolecular disulfide bonds, exposing free thiol groups that facilitate subsequent intermolecular crosslinking [22]. The resulting nanoparticles, characterized by homogeneous and spherical morphology, achieve optimal molecular assembly following incubation in 2-(N-morpholino) ethanesulfonic acid (MES) buffer (pH 4.5) at 37 °C. When evaluating drug-loading efficiency (LE) and encapsulation efficacy (EE), the Min-NP formulation demonstrates a highly controlled, sustained-release profile over a 24 h period. This temporal regulation critically prevents premature payload release within the systemic circulation, thereby exponentially enhancing the bioavailability of the therapeutic agent at the targeted tissue site [125].

In parallel, polymeric–metal hybrid systems constitute another major subclass of biomimetic antifungal nanocarriers, in which chitosan-based architectures are frequently employed as versatile templates for nanoparticle stabilization and fungal membrane interaction. Gold nanoparticle–chitosan hybrids functionalized with bioactive ligands, such as tyrosol (Chi-TY-AuNPs), should therefore be interpreted as a representative model within a broader class of cationic metal–polymer nanosystems. These platforms collectively demonstrate how surface charge modulation, ligand functionalization, and nanoscale size control can be strategically integrated to enhance penetration into fungal biofilms and disrupt membrane integrity. Importantly, the mechanistic insights derived from such systems are not unique to a single formulation but are broadly transferable to other chitosan- and metal-based hybrid nanocarriers. Chitosan, a cationic polysaccharide, functions dually within nanoparticle synthesis: it serves as both a reducing and stabilizing agent, while its abundant primary amine groups facilitate intense electrostatic interactions with the negatively charged fungal cell membrane, potentially augmenting cellular internalization. Chitosan–gold nanoparticles synthesized in situ and functionalized with the quorum-sensing (QS) molecule tyrosol (Chi-TY-AuNPs) exhibit an average hydrodynamic diameter of 10.34 nm [25]. This ultra-small spherical morphology capitalizes on the enhanced permeability and retention (EPR) effect, ensuring deep penetration into the infection microenvironment [126]. The surface plasmon resonance (SPR) characteristics of the embedded gold nanoparticles, verifiable via UV–visible spectroscopy with distinct absorption peaks at 531 nm, confirm robust colloidal stability. They boast a high drug-loading efficiency of 46.08%, and these systems mimic the membrane-fusion dynamics of natural exosomes. Based on current in vitro assays, their polycationic surfaces (manifesting a zeta potential of +45.5 mV) are proposed to induce direct physical perturbation and subsequent pore formation within the fungal membrane. Translating these controlled observations to clinical reality will necessitate rigorous in vivo profiling to ensure that such membrane-disruption mechanisms operate effectively amidst competing host tissue barriers and innate immune surveillance. Furthermore, the expanding armamentarium of nanocarriers extends beyond protein and chitosan-based architectures to encompass next-generation lipidic nanovesicles, including ufasomes (unsaturated fatty acid vesicles), ethosomes (vesicles with high ethanol concentrations), and nanostructured lipid carriers (NLCs) [127]. For instance, the co-delivery of fluconazole with active monoterpenes via NLCs (yielding particle sizes of approximately 144.5 nm and zeta potentials of −23.5 mV) has demonstrated profound synergistic antifungal efficacy. Exosome-inspired flexible vesicles, particularly transferosomes and spanlastics, incorporate edge activators into conventional liposomal frameworks (e.g., *AmB*isome) and possess remarkable ultrastructural deformability [40]. This elasticity permits them to squeeze through intercellular spaces much smaller than their own diameter, facilitating deep dermal penetration and systemic absorption via topical administration routes. Such highly versatile, multi-compartmental biomimetic carriers can be rationally engineered to bypass specific Candida resistance mechanisms, effectively overcoming the overexpression of efflux pumps, target-site gene mutations, and the formidable physical barrier imposed by the EPS matrix [72]. Nevertheless, the structural complexity of exosome-inspired and erythrocyte-cloaked carriers introduces significant technical risks. The precision required to maintain the ‘stealth’ corona during industrial scale-up often results in low yields and high production costs. Simplifying these architectures without sacrificing their biological precision remains a primary goal for the next generation of biomimetic design. Taken together, these selected examples should not be interpreted as isolated case-specific findings, but rather as illustrative representatives of broader design principles governing biomimetic antifungal nanocarriers. The observed physicochemical and biological behaviors are therefore best understood within a unified framework of structure–function relationships that spans protein-, polymer-, and lipid-based nanosystems. Accordingly, exosome-inspired nanocarriers should no longer be viewed solely as structural mimics of endogenous vesicles, but rather as programmable biological delivery systems whose therapeutic versatility extends from antifungal drug transport to targeted gene regulation at the post-transcriptional and genomic levels.

While currently approved lipidic formulations, such as liposomal amphotericin B (AmBisome^®^), have successfully mitigated the severe acute toxicity associated with free antifungal agents, they primarily function as passive delivery vehicles [128]. In contrast, next-generation biomimetic and exosome-inspired nanocarriers offer the distinct clinical advantage of active biological engagement. By incorporating surface-programmed immune evasion and specific molecular targeting, these advanced architectures are designed to actively penetrate dense EPS matrices, transcending the passive accumulation limitations of first-generation liposomes to effectively eradicate deep-seated, multidrug-resistant biofilms [129]. Despite the profound biological advantages of MSC-derived exosomes, their clinical translation is severely impeded by the technical challenges of large-scale manufacturing. Current isolation and purification techniques, such as ultracentrifugation and size-exclusion chromatography, are inherently low-yield and labor-intensive, and they struggle to completely separate target vesicles from contaminating protein aggregates [122]. Furthermore, the inherent biological heterogeneity of the parent cells inevitably leads to significant batch-to-batch variability in vesicle size, surface marker expression, and functional behavior, making it exceedingly difficult to achieve the strict physicochemical standardization required by GMP frameworks.

Beyond isolation and purification, achieving high and reproducible loading efficiencies remains a formidable engineering hurdle. Traditional passive and active payload encapsulation methods (such as electroporation, sonication, or extrusion) frequently result in suboptimal therapeutic concentrations or inadvertently compromise the structural integrity of the delicate exosomal lipid bilayer. Overcoming these industrial barriers necessitates the rapid development of automated, continuous-flow microfluidic technologies capable of ensuring the structural preservation, high-yield production, and uniform standardization essential for the commercial viability of exosome-based antifungal platforms [130].

## 6. Biocompatibility and Cytotoxicity Profiles

A primary translational bottleneck in antifungal nanomedicine is not merely efficacy against pathogenic fungi, but the persistent challenge of achieving this effect without compromising host cellular integrity. Accordingly, current evaluation frameworks increasingly prioritize the systematic assessment of nanocarrier–host interactions, with cytotoxicity readouts such as LDH release, metabolic activity assays, and in vivo biochemical markers serving as comparative indicators across different nanoplatforms [131]. The dose-limiting nephrotoxicity, hepatotoxicity, and hemotoxicity associated with conventional therapies, most notably the polyene amphotericin B, result in devastating cellular damage to renal tubular epithelial cells [132]. In the rigorous preclinical evaluation of nanocarrier biocompatibility, the quantification of LDH leakage has been established as a critical, high-fidelity biomarker. LDH, a stable cytoplasmic enzyme, is rapidly released into the extracellular milieu upon disruption of the plasma membrane [133]. Next-generation nanocarrier systems are structurally optimized to restrict off-target interactions, thereby mitigating unspecific membrane degradation and virtually eliminating pathological LDH release.

Within this context, LDH leakage assays and Cell Counting Kit-8 (CCK-8)-based viability measurements are not specific to individual formulations but rather constitute standard benchmarking tools for assessing membrane integrity and mitochondrial metabolic activity across diverse nanocarrier classes. These assays collectively enable cross-platform comparison of cytocompatibility between lipidic, polymeric, and inorganic systems under standardized experimental conditions. The cytotoxicity and overarching safety profiles of minocycline encapsulated within biomimetic Min-NPs have been subjected to exhaustive toxicological scrutiny. In vitro CCK-8 cell viability assays conducted on mammalian HEK 293T cell lines reveal that even at extremely high exposure concentrations up to 280 µg/mL, Min-NPs preserve nearly 100% cellular viability without precipitating any statistically significant elevation in LDH secretion [134]. Against this broader methodological background, albumin-based nanocarriers such as Min-NPs can be considered representative examples of protein-derived systems that consistently demonstrate favorable biocompatibility profiles in mammalian cell models and animal studies, thereby exemplifying the general safety potential of protein scaffolds rather than constituting an isolated therapeutic exception. This superior in vitro safety is seamlessly mirrored in complex in vivo environments. In systemic candidiasis models using cyclophosphamide-immunosuppressed BALB/c mice inoculated with *C. albicans* via the lateral tail vein, biochemical analyses corroborate the inert nature of the carrier. Similarly, serum biomarkers including ALT, BUN, and creatinine are widely employed as systemic indicators of hepatic and renal tolerance in nanomedicine studies, allowing for standardized assessment of off-target toxicity across different antifungal nanocarrier platforms in immunocompromised infection models. Following seven days of continuous intraperitoneal administration of a Min-NP and fluconazole combinatorial regimen, serum concentrations of the primary hepatic biomarker ALT (alanine aminotransferase), alongside renal functional indicators BUN (blood urea nitrogen) and CRE (creatinine), stabilize at levels strictly equivalent to those of healthy control cohorts (*p* > 0.05) [135]. Moreover, meticulous histopathological examinations of renal tissues utilizing Hematoxylin-Eosin (H&E) and Periodic Acid-Schiff (PAS) staining significantly confirm that the administered nanoparticle dosages inflicted no cellular necrosis, tubule dilation, or inflammatory infiltration within the renal parenchyma [136]. Importantly, across all nanocarrier classes, cytocompatibility is ultimately governed by a balance between targeted fungal membrane disruption—often mediated through ergosterol-selective interactions—and the preservation of mammalian membrane integrity, which is enriched in cholesterol and therefore exhibits differential susceptibility to nanoparticle-induced perturbation.

**Figure 3:**
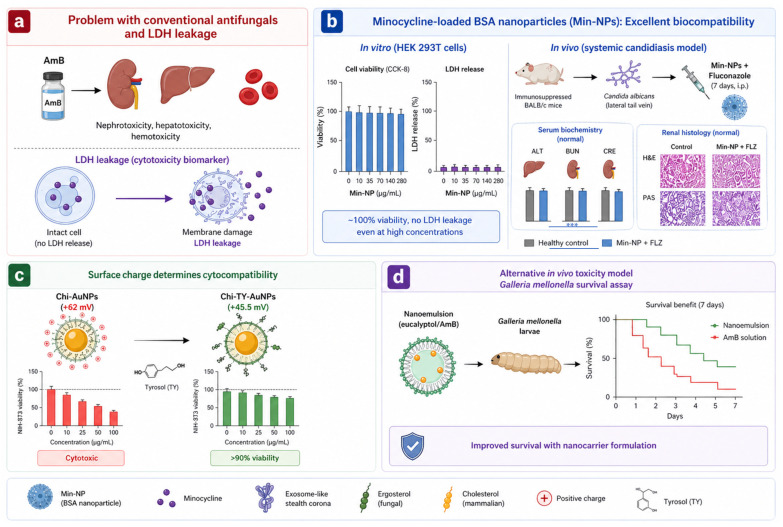
Biocompatible nanocarriers enable antifungal efficacy without host toxicity. (**a**) Conventional antifungals induce dose-limiting toxicity associated with membrane damage and LDH release. (**b**) Minocycline-loaded BSA nanoparticles (Min-NPs) exhibit high biocompatibility, maintaining cell viability and normal liver/kidney biomarkers in vitro and in vivo. For the in vivo serum biochemistry analysis, statistical significance was evaluated by comparing the nanoparticle-treated group (Min-NP + FLZ) directly against the healthy control group (*** denotes *p* < 0.001). (**c**) Surface charge modulation reduces cytotoxicity, as charge-balanced nanoparticles show improved cellular tolerance. (**d**) Rationally designed nanocarriers enhance survival in infection models by selectively targeting fungal membranes while sparing mammalian cells.

Conversely, the cytocompatibility of hybrid inorganic nanoparticles, such as those comprising chitosan and gold, is strictly dictated by their surface charge dynamics. Naked gold nanoparticles reduced directly by chitosan (Chi-AuNPs) exert dose-dependent cytotoxicity on NIH-3T3 murine fibroblast cell lines, a phenomenon directly attributable to their excessively high cationic surface charge (+62 mV), which indiscriminately disrupts mammalian lipid bilayers [137]. However, the rational stoichiometric integration of tyrosol (TY) to yield the Chi-TY-AuNPs formulation significantly mitigates this toxicity. By neutralizing and balancing the net surface potential to +45.5 mV, cellular tolerance is vastly improved, maintaining a biocompatibility threshold of >90% cell survival even at aggressive therapeutic concentrations [138]. Furthermore, alternative in vivo toxicity models, such as the *Galleria mellonella* survival assay utilized for testing eucalyptol/*AmB* nanoemulsions, consistently demonstrate that rationally designed lipidic and polymeric nanocarriers fundamentally protect host tissues [41]. The capacity of these systems to orchestrate specific, targeted assaults solely against fungal membranes—without triggering LDH leakage or mitochondrial dysfunction in mammalian cells—is fundamentally rooted in their ability to exploit the biochemical dichotomy between mammalian cholesterol and fungal ergosterol [139]. By cloaking the API within an exosome-like “stealth” corona, nanocarriers effectively evade macrophage phagocytosis, thereby facilitating pinpoint therapeutic delivery devoid of collateral host toxicity [140] (Figure 3). Collectively, these toxicity evaluation strategies underscore that the clinical translation of antifungal nanomedicines depends not on isolated improvements in individual formulations, but on the establishment of shared design principles that ensure selective antifungal activity while maintaining systemic biocompatibility across diverse biological models.

While cationic nanocarriers are often utilized to improve interaction with negatively charged fungal membranes or biofilms, it is well established that highly cationic nanoparticles are inherently more toxic to host tissues than their anionic or neutral counterparts. This dose-limiting toxicity arises from non-specific electrostatic binding to mammalian cell membranes, leading to membrane destabilization and LDH leakage. As demonstrated in Figure 3c, maintaining an excessively high positive charge (e.g., +62 mV) results in significant cytotoxicity. Therefore, precise modulation of the surface charge—reducing the zeta potential to a more balanced state (+45.5 mV)—is a critical design parameter to achieve a therapeutic window that preserves host cell viability (>90%) while maintaining antifungal efficacy.

## 7. Future Perspectives

Invasive and therapeutically recalcitrant Candida infections currently stand at the forefront of global public health threats within clinical microbiology, driven by alarmingly high morbidity trajectories and a systematic failure of conventional antifungal arsenals. The clinical efficacy of the three primary antifungal pillars—azoles, polyenes, and echinocandins—is being progressively nullified by a confluence of evolutionary adaptations: the impenetrable physical armor of the biofilm extracellular polymeric matrix (EPS), highly specific target enzyme mutations, the massive overexpression of drug efflux pumps, and the insidious persistence of metabolically dormant cellular sub-populations [141]. Synthesizing over two decades of intense structural and functional innovation, nanomedical strategies have forged a highly sophisticated, multi-dimensional toolkit engineered to significantly bypass these formidable resistance barriers at the molecular level [142]. The most formidable technical bottlenecks impeding industrial realization include the rigorous alignment of complex synthesis protocols with pharmaceutical GMP standards, the mitigation of intra-batch and inter-batch variability, and the prohibitive financial costs associated with standardizing intricate nano-architectures [143]. The successful commercial scale-up of multi-component hybrid systems and heavily functionalized biomimetic carriers relies intrinsically on maintaining thermodynamic and kinetic stability over extended shelf lives.

Despite the overwhelming optimism surrounding biomimetic and hybrid nanocarriers in the current literature, a critical evaluation reveals significant translational hurdles that cannot be overlooked. The vast majority of reported successes are confined to highly controlled, optimized in vitro environments that significantly fail to replicate the dynamic shear stresses, protein corona formation, and immunological complexities of the in vivo infection microenvironment [144]. While in vitro LDH release assays and initial murine models demonstrate acceptable short-term cytocompatibility for structures like Min-NPs or chitosan–gold hybrids, the long-term systemic accumulation and potential off-target toxicities of inorganic metallic cores remain concerningly under-investigated [145]. Furthermore, the structural complexity that provides these exosome-inspired systems with their “stealth” properties paradoxically acts as their greatest limitation in clinical translation. Future research must decisively pivot away from merely cataloging new nanostructures with incremental efficacy gains, but instead, the focus must shift toward standardizing toxicity protocols, ensuring batch-to-batch reproducibility, and prioritizing biodegradable, single-component platforms that offer a realistic, scalable pathway [146].

**Figure 4:**
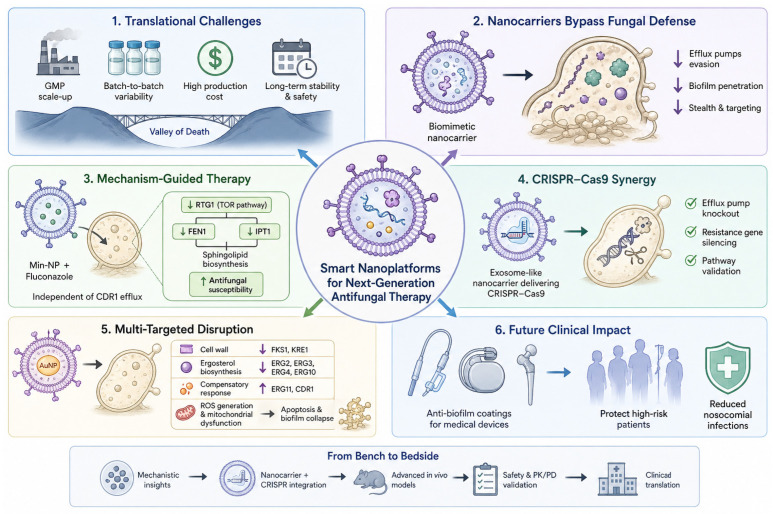
Mechanism-guided antifungal nanoplatforms. Biomimetic nanocarriers enhance drug delivery and bypass fungal defenses. Integrated with CRISPR–Cas9, they reveal and target key resistance pathways (e.g., TOR signaling and lipid biosynthesis), inducing ROS-mediated damage and biofilm collapse, and enabling multi-targeted antifungal therapy. In panel 5, downward arrows (e.g., ↓FKS1) explicitly denote transcriptional downregulation of the specified genes, while upward arrows denote upregulation.

In a highly parallel mechanistic revelation, transcriptomic profiling of the effects of Chi-TY-AuNPs on *N. glabrata* provides critical foresight into future nanobiotechnological applications. These “smart” biomimetic constructs exert significant transcriptional repression on genes crucial for cell wall integrity and membrane fluidity, specifically glucan synthase (FKS1), cell wall proteins (KRE1), and the entire ergosterol biosynthetic cascade (ERG2, ERG3, ERG4, ERG10) [147]. Although the fungal organism attempts to mount a compensatory defense by regulating ERG11 and CDR1 to restabilize its membrane architecture, this effort is fatally short-circuited by the rapid generation of intracellular ROS induced by the nanoparticles [148]. In highly controlled laboratory models, these ‘smart’ biomimetic constructs have been shown to exert significant transcriptional repression on genes crucial for cell wall integrity and membrane fluidity. The resultant nanoparticle-induced oxidative stress is hypothesized to trigger catastrophic mitochondrial dysfunction, culminating in irreversible apoptosis and the subsequent structural collapse of the biofilm architecture [149]. However, these sweeping mechanistic conclusions are currently derived largely from in vitro settings; robust in vivo evidence is urgently needed to confirm if these exact apoptotic and structural degradation pathways occur reliably within the complex physiological landscape of a living host (Figure 4) [150].

The clinical translation of advanced hybrid therapeutic platforms—particularly those integrating small-molecule antifungal agents, exosome-inspired biological carriers, and gene-editing technologies such as CRISPR-Cas9—presents substantial regulatory challenges. Existing regulatory frameworks established by agencies such as the FDA and EMA were primarily developed for conventional single-component therapeutics and often struggle to appropriately classify these multifunctional systems within existing drug, biologic, or medical device categories [151]. As a result, such platforms are frequently assessed as complex combination products, requiring comprehensive characterization of each individual component, including the nanocarrier, biological scaffold, and genetic cargo, as well as their collective pharmacological and toxicological interactions in vivo. This regulatory uncertainty can significantly extend development timelines, increase approval costs, and elevate the risks associated with crossing the translational “Valley of Death” between preclinical innovation and clinical implementation.

Beyond host biocompatibility, the downstream environmental toxicity and ecological impact of metallic nanoparticles (such as silver and gold) following biomedical use and patient excretion warrant critical consideration. Unlike biodegradable lipid- or protein-based carriers, non-biodegradable inorganic nanostructures may persist in municipal wastewater treatment systems and accumulate within aquatic ecosystems. Their intrinsic antimicrobial properties—mediated by sustained metal ion release and continuous generation of ROS—can indiscriminately disrupt environmental microbial communities and pose significant toxicity risks to aquatic organisms [152]. Consequently, the future of antifungal nanomedicine should align with the principles of eco-pharmacovigilance by prioritizing highly biodegradable platforms, such as protein-based scaffolds or exosome-inspired biological carriers, to minimize hazardous environmental accumulation after disposal.

To accurately contextualize the translational landscape of antifungal nanomedicines, it is critical to distinguish between purely proof-of-concept technologies and those approaching clinical readiness. Highly complex architectures—such as CRISPR-Cas9-loaded exosome-inspired vesicles and erythrocyte-cloaked inorganic hybrids (e.g., tyrosol-functionalized gold nanoparticles)—remain firmly in the early preclinical, proof-of-concept stage due to unresolved challenges in GMP scale-up, structural fragility, and long-term biosecurity profiling [153]. Conversely, lipid-based systems, particularly SLNs and nanoemulsions formulated for topical applications, have already bypassed several systemic regulatory barriers and advanced into early human clinical trials. Similarly, albumin-based nanocarriers (such as Min-NPs) are rapidly approaching clinical readiness for systemic application, leveraging the well-established pharmaceutical precedent, high biocompatibility, and highly scalable manufacturing protocols associated with protein scaffolds [154].

Furthermore, the fundamental mechanism that grants metallic nanoparticles their antifungal efficacy—the rapid generation of ROS—acts as a double-edged sword. While targeted ROS storms effectively collapse fungal biofilms and trigger mitochondrial dysfunction in Candida, uncontrolled or chronic oxidative stress can precipitate severe collateral damage to mammalian cells [155]. Prolonged tissue exposure to non-degradable metallic cores can induce lipid peroxidation, mammalian DNA damage, and the activation of pro-inflammatory pathways. This chronic physical presence often provokes sustained host inflammatory responses, hyperactivating macrophages and potentially leading to localized granuloma formation or systemic cytokine dysregulation. While charge-balancing strategies (such as tyrosol functionalization) successfully mitigate acute cytotoxicity and membrane disruption, establishing the ultimate clinical viability of metallic nanocarriers requires exhaustive, longitudinal in vivo pharmacokinetic tracking to definitively rule out chronic oxidative toxicity and immunogenicity [156].

Looking forward, the precise engineering of exosome-inspired, highly biocompatible nanoplatforms mapped explicitly to these genomic vulnerabilities will guarantee spatiotemporally controlled drug release directly within the infectious nidus. Furthermore, the translation of these nanotechnologies into macro-scale medical applications—such as the integration of polymeric nanofibers and anti-fouling chitosan/gold nanocomposites into the surface matrices of catheters, pacemakers, and orthopedic implants—promises a highly effective strategy to preemptively inhibit and significantly diminish nosocomial biofilm colonization at the source [157]. Extended longitudinal pharmacokinetic and pharmacodynamic models will ultimately dictate the speed at which these advanced therapeutics are integrated into clinical protocols, offering renewed hope for severely immunosuppressed and high-risk patient demographics.

## 8. Conclusions

Synergistic nanoparticulate formulations, rationally designed to counteract fungal resistance mechanisms and improve the therapeutic index, have demonstrated significant preclinical potential. For instance, the targeted co-delivery of agents such as minocycline and fluconazole via albumin nanoparticles (Min-NPs) has shown substantial synergistic activity against resistant *Candida* isolates in vitro (FICI ≤ 0.5) and has successfully reduced renal fungal burdens while extending host survival in preliminary murine models [22]. Furthermore, early in vivo assessments suggest that biomimetic, exosome-inspired carriers can maintain stable hepatic and renal biomarkers, indicating a potentially improved acute safety profile compared to conventional therapies [158]. However, characterizing these platforms as a definitive clinical solution remains premature. The transition across the translational “Valley of Death” is currently hindered by substantial practical and biological barriers. These challenges include the profound engineering complexities of GMP-compliant scale-up, the uncharacterized long-term toxicity of hybrid inorganic systems, and the unpredictable pharmacokinetic behavior of complex nanocarriers when subjected to dynamic host immune responses and physiological shear stress.

To fully realize the therapeutic value of next-generation nanomedicines, future research must prioritize rigorous, long-term in vivo validation using advanced infection models that accurately simulate the human microenvironment. Emphasizing comprehensive chronic immunogenicity profiling and the development of simplified, highly reproducible, and biodegradable architectures will be critical. Addressing these translational bottlenecks is essential for nanotechnology-driven strategies to safely and effectively advance from promising laboratory innovations to accessible clinical standards in the management of invasive candidiasis.

## Figures and Tables

**Table 1:** Comparative efficacy and biofilm interactions of nanosystems against priority fungal species.

Fungal Species	Matrix/Biofilm Characteristics	Notable Nanosystems and Compounds Evaluated	Performance and Therapeutic Impact	References
***C. albicans***	Matrix is highly dense, rich in eDNA, polysaccharides (β-1,3-glucan), and structural glycoproteins.	Tyrosol-Functionalized Gold NPs; Solid Lipid Nanoparticles (SLNs) loaded with Fluconazole.	Promotes deep biofilm degradation, enhances intracellular drug accumulation, and downregulates key biofilm-associated genes.	[25,26]
***C. auris***	Forms robust, highly adherent biofilms prone to rapid multidrug resistance and persistence on medical surfaces.	Nanocarriers combining traditional Antifungal Drugs with Monoterpene Phenols (e.g., Carvacrol, Linalool).	Induces significant disruption of fungal membrane integrity, potentially circumventing active efflux pumps (ABC/MFS) and restoring fungicidal activity.	[24,27]
***N. glabrata***	Exhibits distinct EPS matrix organization with high protein-to-carbohydrate ratios and less fibrillar glucan structures.	Albumin Nanoparticles (BSA scaffolds) co-delivering Minocycline and Fluconazole.	Successfully eradicates azole-resistant strains at clinically safe doses by synergistically overwhelming complex fungal defense networks.	[22,28]
***C. tropicalis***	Known for rapid resistance acquisition and high secretion of viscous matrix components during standard therapies.	Nanosystems co-delivering Minocycline and Fluconazole; Nanostructured Lipid Carriers (NLCs).	Synergistic action effectively augments azole efficacy against drug-resistant phenotypic variants and suppresses active efflux.	[22,29]
***C. parapsilosis***	Characterized by variable lipid compositions, unique matrix structures, and large, irregular cell cluster formations.	Broad-spectrum metallic nanocarriers (e.g., Biogenic Silver and Copper NPs).	Overcomes species-specific structural barriers through non-specific cell wall/DNA damage and rapid generation of reactive oxygen species (ROS).	[20,30]

**Table 5:** Candida resistance barriers and corresponding nano-solutions.

Resistance Barrier	Biological/Evolutionary Basis	Nanotechnological Solution	Impact on Clinical Outcomes	Ref.
**Dense EPS Matrix**	beta-glucan\alpha-mannan, and eDNA shield.	Small particle size (<100 nm); enzymatic or mucoadhesive functionalization.	Bypasses physical sequestration; delivers drugs to deep sessile populations.	[102]
**Efflux Pump Overexpression**	Active extrusion via ABC (*CDR1/2*) and MFS (*MDR1*) transporters.	High localized drug delivery; co-encapsulation of pump inhibitors.	Overwhelms efflux capacity; restores drug sensitivity in MDR strains.	[103]
**Persister Cells**	Metabolically dormant, tolerant subpopulations.	Intrinsic metallic NP toxicity (ROS); sustained-release lipid carriers.	Effectively target and deplete dormant reservoirs independent of active metabolism; reduces relapse.	[104]
**Systemic Host Toxicity**	Off-target damage to renal and blood cells (*AmB*).	Biomimetic membrane coatings; targeted “stealth” carriers.	Evades RES clearance; dramatically reduces LDH release and systemic side effects.	[105]
**Target Site Mutations**	Structural changes in target enzymes (e.g., *ERG11*).	Multi-target metallic NP action; delivery of novel payloads (Olorofim).	Overcomes specific target resistance through non-specific cell wall/DNA damage.	[106]

## Data Availability

No new data were created or analyzed in this study. Data sharing is not applicable to this article.

## Abbreviations

The following abbreviations are used in this manuscript:

ABC	ATP-binding cassette
Ag	Silver
AgNPs	Biogenic Silver NPs
ALT	Alanine aminotransferase
*AmB*	Amphotericin B
API	Active pharmaceutical ingredient
Au	Gold
BSA	Bovine serum albumin
BUN	Blood urea nitrogen
CCK-8	Cell viability assays
CFU	Colony-forming unit
Chi-AuNPs	Naked gold nanoparticles reduced directly by chitosan
Chi-TY-AuNPs	Tyrosol-functionalized gold NPs
CRE	Creatinine
Cu	Copper
DTT	Dithiothreitol
eDNA	Extracellular DNA
EE	Encapsulation efficacy
EPR	Enhanced permeability and retention
EPS	Extracellular polymeric substance
FICI	Fractional Inhibitory Concentration Index
FLC	Fluconazole
GMP	Good Manufacturing Practice
H&E	Hematoxylin-Eosin
IFIs	Invasive fungal infections
LDH	Lactate Dehydrogenase
LE	Loading efficiency
MDR	Multidrug-resistant
MES	2-(N-morpholino) ethanesulfonic acid
MFS	Major Facilitator Superfamily (MDR1)
Min-NPs	Albumin-minocycline nanoparticles
MPS	Mononuclear phagocyte system
N/A	Not applicable
NAC	Non-albicans Candida
NE	Nanoemulsions
NLCs	Nanostructured lipid carriers
nm	Nanometer
NPs	Nanoparticles
PAS	Periodic Acid-Schiff
PDI	Polydispersity index
qRT-PCR	Quantitative reverse transcription PCR
QS	Quorum-sensing
RES	Reticuloendothelial system
RNPs	Ribonucleoproteins
ROS	Reactive oxygen species
SLN	Solid lipid nanoparticles
SPR	Surface plasmon resonance
TOR	Target of rapamycin
TY	Tyrosol
UV	Ultraviolet/UV–visible spectroscopy
